# Circulating cancer stem cell markers in breast carcinomas: a systematic review protocol

**DOI:** 10.1186/s13643-017-0660-y

**Published:** 2017-12-19

**Authors:** Maryam Mansoori, Zahra Madjd, Leila Janani, Arezoo Rasti

**Affiliations:** 1 Department of Molecular Medicine, Faculty of Advanced Technologies in Medicine, Iran University of Medical Sciences (IUMS), Tehran, Iran; 2 Department of Molecular Medicine, Oncopathology Research Center, Faculty of Advanced Technologies in Medicine, Iran University of Medical Sciences, Tehran, Iran; 3 Department of Biostatistics, School of Public health, Iran University of Medical Sciences (IUMS), Tehran, Iran; 4 Oncopathology Research Centre, Iran University of medical Sciences (IUMS), Tehran, Iran

**Keywords:** Breast cancer, Cancer stem cell (CSC), Circulating tumor cell (CTC), Circulating cancer stem cell (CCSC), Marker

## Abstract

**Background:**

Breast cancer is one of the most common types of cancer in women worldwide. Recent studies have provided strong support for the cancer stem cell (CSC) hypothesis, which suggests that many cancers, including breast cancer, are driven by a subpopulation of cells that display stem cell-like properties. The hypothesis that a subpopulation of circulating tumor cells (CTCs) possesses many CSC-like hallmarks is reinforced by the expression of related molecular markers between these two cell populations. The aim of this study is to systematically review primary studies and identify circulating CSC markers in breast cancer patients.

**Methods and design:**

Relevant observational studies evaluating the expression of circulating breast cancer stem cell markers through October 31, 2016, will be searched in PubMed, SCOPUS, Embase, ISI Web of Science, and Google Scholar with no restriction on language. Full copies of articles identified by the search and considered to meet the inclusion criteria will be obtained for data extraction and synthesis. Two quality assessment tools will be used for evaluating observational studies like case control, which are the Hoy et al. suggested tool and Newcastle-Ottawa Scale (NOS), respectively. Publication bias will be assessed by funnel plots or Egger’s test (i.e., plots of study results against precision), and data synthesis will be performed using Stata software (Stata Corp V.12, TX, USA).This systematic review will be reported according to the Preferred Reporting Items for Systematic Reviews and Meta-Analyses (PRISMA).

**Discussion:**

Detecting cancer stem cells in blood will help clinicians to monitor cancer patients by obtaining as many samples as needed with a non-invasive method and in any stages; it is not possible to repeat sampling on working on tissue samples. By identifying cancer stem cells early in blood, it will be possible to distinguish metastasis in early stages.

**Systematic review registration:**

CRD42016043810

**Electronic supplementary material:**

The online version of this article (10.1186/s13643-017-0660-y) contains supplementary material, which is available to authorized users.

## Strengths and limitations of this study

- This systematic review will, for the first time, evaluate circulating cancer stem cell markers in breast carcinomas without placing restrictions on language.
- Since there is no specific marker for detecting circulating cancer stem cells in different subtypes of breast cancer, we aim to define the best marker(s) for identification of breast cancer stem cells in the blood.
- Though the majority of cancer stem cell studies focus on tissues, identification of cancer stem cells in the blood can be applicable in diagnosis, monitoring of patients, and targeted therapy of tumors.
- The study screening, data extraction, and assessment of risk of bias of the current study will be conducted independently by two researchers.
- There is high heterogeneity of cancer stem cell markers in the circulating blood of breast cancer patients.
- This review will be limited by the quality and heterogeneity of the primary studies.

## Background

Breast cancer is the sixth most common cause of cancer-related deaths worldwide and is the second most common cause of cancer death in women. The incidence of breast cancer is highest in high-income countries, accounting for more than half of all breast cancer cases. Although breast cancer is less common in women living in low-income countries, the age-adjusted incidence is increasing, and the rates of increase are often greater in these countries [1].

Circulating tumor cells (CTCs) are a distinct population of cancer cells that have detached from the primary tumor and intravasated into the blood, a necessary step in forming distant metastases. It is believed that tumor infiltration of blood vessels at the primary tumor site is responsible for the dissemination of tumor cells and the formation of metastatic lesions in distant organs. This suggests that they may share the hallmarks of cancer stem cells (CSCs), as CSCs have the ability to give rise to new tumors. Due to their role in metastasis, CTCs can be considered the progenitors of relapse and direct contributors to the development of metastasis [2, 3].

Many techniques have been developed and are under continuous improvement to enhance the efficacy of CTC isolation and characterization. They have been proven useful in detecting minor subgroups of cells present in the primary tumor that may contribute to treatment resistance or relapse. Hence, detecting and characterizing CTCs may be a useful tool in treating solid malignancies [4].

Recent studies have provided strong support for the cancer stem cell hypothesis, which suggests that many cancers, including breast cancer, are driven by a subpopulation of cells that display stem cell properties. These cells may mediate metastasis and, by virtue of their relative resistance to chemotherapy and radiation, contribute to treatment relapse [5].

Various markers are used to identify the unique subpopulation of breast cancer cells with stem cell properties. Whether these markers are expressed in all breast cancers, identify the same population of cells, or equate to therapeutic response is controversial. The relationship of breast cancer stem cells with clinical parameters will require the identification of specific markers for the individual cancer patient [6].

CTCs and CSCs have been proposed as valuable tools for the detection and characterization of disease and individualization of therapy for multiple solid tumors. A focused and prospective validation of the clinical utility of detecting these cells is still needed, but results suggest that they may add great benefit for the early detection and personalization of therapy [7].

One well-defined method for the identification of putative breast cancer stem cells (BCSCs) is flow cytometry [8]. For the first time, breast cancer-initiating cells were isolated based on the expression of unique cell surface antigens, which include epithelial specific antigen (ESA) and CD44, but not CD24 [9]. Subsequent studies demonstrated that the CD44^+^ and CD24^−^ phenotype can define a population enriched for BCSCs. Other cell surface markers, like CD49f and CD133, can, when combined with CD44^+^ and CD24^−^, be used to identify BCSCs in different breast cancer subtypes [10, 11]. BCSCs can also be isolated using the Aldefluor assay based on aldehyde dehydrogenase (ALDH) activity [12]. The EpCAM^+^, CD24^−^, CD44^+^, and ALDH^+^ populations across different subtypes of breast cancers represent anatomically distinct BCSCs with respective EMT (epithelial-to-mesenchymal transition) and MET (mesenchymal-to-epithelial transition) gene expression profiles. These populations dynamically transition between the mesenchymal and epithelial states reflective of their normal counterparts in the mammary epithelial hierarchy [13].

Although identification of these markers in tissue may be meaningful clinically, in practice, it is not possible to take repeated tissue samples from the breast. However, it is possible to take peripheral blood samples multiple times. Due to the high heterogeneity of breast cancers and the diversity of BCSC markers, many studies suggest using a panel of markers for the identification of BCSCs. However, previous attempts to discover appropriate BCSC markers have not been successful.

In previous studies, different markers of tumor cells, including EPCAM, CD44, CD24, ALDH1, CD133, and PIWIL2, found in the blood of breast cancer patients were evaluated to determine whether it was possible to use circulating BCSCs for patient monitoring, prognosis, diagnosis, and response to therapy [14–23]. To date, several researchers have published their data on stem cell markers in CTCs in patients with breast cancer, but there is no systematic review on identifying markers of circulating BCSCs.

## Methods/design

### Objectives

The objective of this review is to provide a systematic review of primary studies to identify the BCSC markers in the circulating blood of breast cancer patients. This review will complement the findings of an existing review published in 2015 [24].

### Review questions

This review of studies should address the following questions:

1. Establish the existence of circulating CSC markers in patients with breast carcinomas
2. Establish the identification of circulating CSC markers in patients with breast carcinomas

## Study design

This review protocol has been published in the PROSPERO international prospective register of systematic reviews (http://www.crd.york.ac.uk/PROSPERO), registration number CRD42016043810.

The Preferred Reporting Items for Systematic Reviews and Meta-Analyses for Protocols 2015 (PRISMA-P 2015) have been used for preparing and reporting the protocol of this systematic review [25]. The methods adopted for this systematic review have been developed in accordance with the guidelines detailed on the PRISMA checklist, and the PRISMA flow diagram will be used to describe the flow of information through the different phases of the systematic review (Additional file 1) [26].

## Criteria for considering studies for the review

### Inclusion criteria

Observational studies (cross-sectional, case-control, and cohort) which assessed circulating CSC markers and identified these markers in breast carcinomas based on defined methods will be included. These methods which are used for CSC identification and isolation are as follows:

1. *Magnetic-activated cell sorting* (*MACS*) is a method for separation of various cell populations depending on their surface antigens (CD molecules). In this method, the super paramagnetic nanoparticles are used to tag the targeted cells in order to capture them inside the column.
2. *Flow cytometry* is a laser- or impedance-based, biophysical technology employed in cell counting and biomarker detection which allows an individual characterization of rare cells like CTCs. It is used for immunophenotyping the cells. It is predominantly used to measure fluorescence intensity produced by fluorescent-labeled antibodies detecting proteins or ligands that bind to specific cell-associated molecules.
3. *Fluorescence-activated cell sorting* (*FACS*) is a specialized type of flow cytometry. It provides a method for sorting a heterogeneous mixture of biological cells into two or more containers, one cell at a time, based upon the attached conjugated antibody to its surface emits specific fluorescent lights which is used for characterization of each cell. It is a useful scientific instrument as it provides fast, objective, and quantitative recording of fluorescent signals from individual cells as well as physical separation of cells of particular interest.
4. *Reverse transcription polymerase chain reaction* (*RT-PCR*) is a variant of polymerase chain reaction (PCR) and is a technique commonly used in molecular biology to detect RNA expression and to measure the quality of the gene expression in cells.
5. *Real-time polymerase chain reaction* (*real-time PCR*), also known as quantitative polymerase chain reaction (qPCR). It monitors the amplification of a targeted DNA molecule during the PCR, i.e., in real-time and not at its end, as in conventional PCR. Real-time PCR can be used quantitatively (quantitative real-time PCR) and semi-quantitatively, i.e., above/below a certain amount of DNA molecules (semi quantitative real-time PCR).
6. *Filtration* is used to enrich CTC which is solely based on the size of the cells.
7. *Density gradient centrifugation and immunocytochemistry* is one way to enrich disseminated tumor cells. Mononuclear cells are isolated using Ficoll and are subsequently spun on glass slides. Visualization of the tumor cells beside the leukocytes is effected by means of immunocytochemistry.
8. *Chip based methods:*
  - *Microfluidics*, *immobilization and in situ hybridization* (*ISH*) uses special chips combining microfluidics and immobilization of CTCs by binding of specific antibodies. The latter chip is used to establish RNA ISH assay to detect and quantify CTCs. ISH is a type of hybridization that uses a labeled complementary DNA, RNA, or modified nucleic acids strand (i.e., probe) to localize a specific DNA or RNA sequence in cells (e.g., CTC-chip, Herringbone Chip).
  - *A novel chip-based platform using size-based separation* separates nucleated cells from whole blood by using size-based separation, then aligns cells in a microfluidic channel using inertial focusing, and subsequently isolates CTCs by means of negative selection (leukocytes depletion) using microfluidic magnetophoresis. This method significantly reduces contamination of the isolated CTCs with undesired hematopoietic cells as well as includes CTCs that have undergone epithelial to mesenchymal transition (EMT) and thus lost epithelial traits.

### Participants

Adult patients with breast cancer, any subtype, will be included.

### Outcomes

The primary outcome will be the expression pattern of circulating CSC markers in breast carcinomas including EPCAM, CD44, CD24, ALDH1, CD133, and PIWIL2. The secondary outcome will be expression pattern of circulating CSC markers in individual subtypes of breast carcinomas.

### Types of studies to be excluded

Studies assessing animal models, reviews, letters, editorials, case reports, and case series studies will be excluded.

## Search methods for identification of studies

### Electronic searches

To identify the relevant studies, we will search the databases PubMed, Web of Science, Embase, Scopus, and Google Scholar (up to October 2016).

### PUBMED search strategy

In the PubMed database, the search syntax will be: Breast AND (Cancer OR Neoplasm OR Carcinoma OR Tumor OR Tumour OR Cancers OR Neoplasms) AND circulating AND (“tumor cell” OR “cancer cell” OR “cancer stem cell” OR “initiating stem cell” OR “tumor initiating cell”) AND (“stemness marker” OR marker OR “surface marker”). The search syntax will be modified in other databases.

### Data extraction (selection and coding)

Two reviewers (M. Mansoori and L. Janani) will independently extract the following information from each study:

1. Study characteristics (author, year of publication, language of publication, country, study design, setting, locations, criteria for sample selection and sample size, diagnostic criteria, outcomes measured, and patient enrolment strategies)

2. Participants’ characteristics (age, gender)

3. Frequency estimates of cancer stem cell marker expression on the surface of circulating tumor cells. Discrepancies between the two reviewers will be resolved by discussion. An independent investigator will be consulted to reach a consensus where there is uncertainty or disagreement between the reviewers.

### Risk of bias (quality) assessment

Two reviewers, LJ and MM, will independently assess the methodological quality of primary studies by a quality assessment tool developed by Hoy et al. [27] and adapted by Werfalli et al. [28]. It will be applied and further adapted, if necessary, to all screened full-text articles in order to assess study quality. The defined questions will be answered and the score of each article will be calculated using this assessment tool. Studies will be graded as low risk, moderate risk, and high risk for scores of 6, 6–8, and > 8, respectively. The Newcastle-Ottawa Scale (NOS) will be used to assess the quality of studies, if the case-control primary studies will be included in the systematic review. This tool was developed as a collaboration between the University of Newcastle, Australia, and the University of Ottawa, Canada, using a Delphi process to define variables for data extraction. Using the tool, each study is judged on eight items, categorized into three groups: the selection of the study groups, the comparability of the groups, and the ascertainment of either the exposure or outcome of interest for case-control or cohort studies, respectively [29]. An independent investigator will be consulted to reach a consensus where there is uncertainty or disagreement between the reviewers.

### Strategy for data synthesis

We will review and present all included studies in two separate tables. The first table will provide details on study quality according to the abovementioned tool. The other table will include study design, patient demographic, and identification of circulating CSC markers in patients with breast carcinomas. The guidelines suggested by the Cochrane Handbook for Systematic Reviews of Interventions (Higgins 2008) will be used for appropriate statistical analysis. Data will be analyzed using Stata software (Stata Corp V.12, TX, USA). Initially, the data will be analyzed using a narrative method. For studies with control group, we planned to use meta-analysis to combine the results. We will present dichotomous outcomes as odds ratios with 95% confidence intervals (CI). Heterogeneity will be evaluated to determine the extent of variation in effect estimates due to heterogeneity rather than chance. The heterogeneity among the primary studies will be evaluated by a visual inspection of the forest plots, *χ* ^2^ test (with significance defined at α-level of 10%) and *I* ^2^ statistic. Categories of heterogeneity will be ≤ 25% low, 26–50% moderate, 51–75% substantial, and ≥ 76% as considerable heterogeneity as defined by Higgins. For low and moderate level of heterogeneity, we will use fixed-effect model, and a random-effects model will be used in situations where the level of heterogeneity is substantial. If there will be a considerable level of heterogeneity and the number of studies will be enough, we will try to identify potential sources of heterogeneity by investigating individual studies and using meta-regression. Publication bias will be assessed by funnel plots and Egger’s test. Sensitivity analysis will be performed according to quality of the studies and differences in countries when appropriate.

### Analysis of subgroups or subsets

Subgroup analysis will also be used when appropriate (for example, according to cancer subtypes and age groups).

## Discussion

This review will summarize the data around different markers of circulating cancer stem cell in breast carcinomas. We will report the results of our review in agreement with the PRISMA statement (PRISMA-P 2015 checklist). We aim to introduce marker(s) to best detect breast cancer stem cells in circulation since there is no existing any other similar study. Detecting cancer stem cells in blood will help clinicians to monitor cancer patients by obtaining as many samples as needed with a non-invasive method and in any stages; it is not possible to repeat sampling on working on tissue samples. By identifying cancer stem cells early in blood, it will be possible to distinguish metastasis in early stages.

To our knowledge, this review will be the first of its kind to address this topic. The results of this study can be applicable in diagnosis, monitoring of patients, and targeted therapy of tumors for breast cancer patients.

## Additional file

Additional file 1: PRISMA-P (Preferred Reporting Items for Systematic Review and Meta-Analysis Protocols) 2015 checklist. (DOC 85 kb)

## Abbreviations

BCSC: Breast cancer stem cell
CCSC: Circulating cancer stem cell
CSC: Cancer stem cell
CTC: Circulating tumor cell
